# Reproductive trait differences drive offspring production in urban cavity‐nesting bees and wasps

**DOI:** 10.1002/ece3.7537

**Published:** 2021-06-22

**Authors:** Marco Moretti, Simone Fontana, Kelly A. Carscadden, J. Scott MacIvor

**Affiliations:** ^1^ Biodiversity and Conservation Biology Swiss Federal Research Institute WSL Birmensdorf Switzerland; ^2^ Nature Conservation and Landscape Ecology University of Freiburg Freiburg Germany; ^3^ Department of Ecology and Evolutionary Biology University of Colorado Boulder CO USA; ^4^ Department of Biological Sciences University of Toronto Scarborough Toronto ON Canada

**Keywords:** cities, fitness, functional diversity, individual‐based trait variation, trait diversity, urban green space

## Abstract

The contrasting and idiosyncratic changes in biodiversity that have been documented across urbanization gradients call for a more mechanistic understanding of urban community assembly. The reproductive success of organisms in cities should underpin their population persistence and the maintenance of biodiversity in urban landscapes. We propose that exploring individual‐level reproductive traits and environmental drivers of reproductive success could provide the necessary links between environmental conditions, offspring production, and biodiversity in urban areas. For 3 years, we studied cavity‐nesting solitary bees and wasps in four urban green space types across Toronto, Canada. We measured three reproductive traits of each nest: the total number of brood cells, the proportion of parasite‐free cells, and the proportion of non‐emerged brood cells that were parasite‐free. We determined (a) how reproductive traits, trait diversity and offspring production respond to multiple environmental variables and (b) how well reproductive trait variation explains the offspring production of single nests, by reflecting the different ways organisms navigate trade‐offs between gathering of resources and exposure to parasites. Our results showed that environmental variables were poor predictors of mean reproductive trait values, trait diversity, and offspring production. However, offspring production was highly positively correlated with reproductive trait evenness and negatively correlated with trait richness and divergence. This suggests that a narrow range of reproductive traits are optimal for reproduction, and the even distribution of individual reproductive traits across those optimal phenotypes is consistent with the idea that selection could favor diverse reproductive strategies to reduce competition. This study is novel in its exploration of individual‐level reproductive traits and its consideration of multiple axes of urbanization. Reproductive trait variation did not follow previously reported biodiversity‐urbanization patterns; the insensitivity to urbanization gradients raise questions about the role of the spatial mosaic of habitats in cities and the disconnections between different metrics of biodiversity.

## Graphical abstract

We studied intraspecific reproductive traits and offspring production of cavity‐nesting bees and wasps across in the city of Toronto, Canada. We found that offspring production was highly positively correlated with reproductive trait evenness and negatively correlated with trait richness and divergence. This suggests that a narrow range of reproductive traits are optimal for reproduction, and the even distribution of individual reproductive traits across those optimal phenotypes could indicate selection for diverse reproductive strategies to reduce competition. We found little changes in reproductive trait variation or offspring production along urbanization gradients.

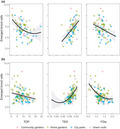

## 1 INTRODUCTION

Urban areas are expanding at an unprecedented speed as the global human population increases. One of the most urgent questions is whether and how urban areas can sustain diverse and resilient biological communities (Barot et al., 2019). So far, research has shown contrasting responses of biodiversity to different aspects of urbanization within and among cities. Patterns vary with taxonomic groups (Concepcion et al., 2017), spatial features and spatial scales (Seto et al., 2010), land‐use legacy of new‐urban areas and the time elapsed since urbanization started (Ramalho et al., 2012), as well as the amount and types of urban green space (UGS) present in cities (Fournier et al., 2020). This contingency makes it crucial to move beyond pattern‐based studies to identify mechanisms that enable biodiverse communities to persist in novel, human‐dominated systems. Namely, understanding the factors that drive species’ reproduction and survival should help to explain variation in biodiversity across an urban landscape.

Partitioning urbanization gradients into their underlying, interacting environmental variables can clarify the factors driving biotic responses. For example, urbanization leads to a myriad of different land uses that collectively can increase environmental heterogeneity in the local urban landscape (Lepczyk et al., 2017; Malkinson et al., 2018). By characterizing urbanization as both the proportion of impervious surface within a defined area and the degree of heterogeneity in the landscape, and by considering the patchwork of UGS types, such as parks, green roofs, home, and community gardens, we might be able to better interpret patterns in species or trait composition (Aronson et al., 2016; Fournier et al., 2020). For animals in urban spaces, multiple environmental variables will provide different types and quantity of feeding and nesting resources with possible consequences for the phenotypes and strategies necessary to guarantee reproductive success (e.g., Zurbuchen et al., 2010).

Focusing on traits rather than species can elucidate mechanisms behind community assembly in cities (Aronson et al., 2016; Brousseau et al., 2018). Studies have typically selected traits theorized to respond to the environmental changes that accompany urbanization; however, in many cases, the relationship of these traits with reproduction and fitness is not tested (Moretti et al., 2017). Moreover, traits are often quantified as mean values for each species obtained from the literature (Schneider et al., 2019). While this approach has its advantages, traits can vary greatly among environments and populations. Trait variation within species can be substantial (Carscadden et al., 2017; Warzecha et al., 2016) and an important determinant of species’ ability to respond to environmental change and impact key ecological processes across scales (Agrawal, 2001; Bolnick et al., 2011; Miner et al., 2005; Stomp et al., 2008). Few studies of urban biodiversity patterns have considered individual‐based traits (as defined by Violle et al., 2007) and their implications for individual offspring production (e.g., Ibanez‐Alamo & Soler, 2010).

Here, we use solitary cavity‐nest provisioning (cavity‐nesting) bee and wasp populations from trap nests as a model system, which allows easy quantification of interactions between bees, wasps, their food objects, and natural enemies (MacIvor, 2017; Staab et al., 2018). These taxa are known to respond to urbanization at local and landscape scales (Pereira‐Peixoto et al., 2014; Sexton et al., 2021). Cavity‐nesting bees and wasps depend on similar nesting conditions (Tscharntke et al., 1998) but occupy separate trophic niches (bees as pollinators and wasps as predators). Comparing the responses to urbanization between bees and wasps will reveal whether similar trends in biodiversity are present, and similar trait‐based mechanisms involved. Here, we focus on three reproductive traits measured at the level of single nest tunnels (hereafter, ‘nesting tubes’) to assess their responses to environmental variables and impacts on offspring production. The three reproductive traits are presented in Box 1.

BOX 1 **Reproductive traits and offspring production**

**Reproductive traits**

1. The first trait is the *total number of brood cells per nest*. This proxy of fecundity is defined as the number of eggs laid by one individual female in one individual nesting tube, similar to ‘clutch size’ described in Moretti et al. (2017). Clutch size, together with other life‐history traits such as age at maturity, voltinism and life span, have strong links to fitness and are expected to be among the most sensitive to environmental stress, making them useful to assess the vulnerability of species to global change. The total number of brood cells per nest should vary depending on the reproductive strategy of individual females and differences among species in the type and number of resources required to provision a single brood cell. That is, trade‐offs between time spent caring for the brood (reproductive traits) and time spent away foraging for pollen and nectar (bees) or prey (wasps) in the surrounding landscape (dispersal traits) (Guerra, 2011) may result in reduced reproductive investment in individuals that have to move long distance to forage (as they may in densely urban areas). For example, *Megachile pugnata* have specialized diets of *Asteraceae* pollen and require leaf cuttings and soil to build their brood cells (Frolich & Parker, 1983). If these resources are scarce near the nest, we expect the number of brood cells of *M. pugnata* to be lower as compared to the more generalist co‐occurring *Megachile* *rotundata* (MacIvor, 2016; Pitts‐Singer & Cane, 2011).
2. The second trait is the *proportion of non‐emerged* *(parasite‐free)* *brood cells per nest*, defined as the number of brood cells that failed to yield an offspring (for reasons other than parasites) divided by the total number of brood cells per nesting tube. This trait represents one component of reproductive failure. Pathogens obtained from flowers during foraging (Batra et al., 1973) can be transferred to developing bees and prevent their emergence. Certain approaches to brood cell construction, such as using mud rather than plant material to seal cells or selecting leaves from plants having antimicrobial properties (MacIvor, 2016), could reduce brood exposure to pathogens. Pathogenic prevalence in bees, and therefore this source of reproductive failure, is known to vary with the types of flowers visited and anthropogenic changes to landscapes (McArt et al., 2014). Wasps are much more poorly studied in this regard, but we expect non‐emerged brood to be in part a result of the juvenile wasp being injured by prey that are semiparalyzed in the nest.
3. Lastly, the third trait is the *proportion of parasite‐free cells per nest* defined as the number of brood cells that were not attacked by parasites, divided by the total number of brood cells per nesting tube. It estimates the proportion of offspring that escaped from one key source of reproductive failure (parasitism). Parasites and natural enemies represent a large source of brood mortality in cavity‐nesting bees (16.6% ± 16.8 average ± *SD* mortality from 111 datasets reviewed in Minckley and Danforth (2019)). Many parasites normally attack when the female is away (e.g., on foraging trips). We speculate that females that must forage longer, if high‐quality or preferred resources are not available nearby, may create a longer window of opportunity for parasites. This idea is supported by a flight cage study of one of our focal species (*Osmia pumila*), which showed that females remained at their nests longer between foraging trips when their specialist cleptoparasite (*Sapyga centrata*) was present Goodell (2003).

**Offspring production**

Offspring production for each nesting site was calculated as the number of successfully emerged brood cells (averaged across single nesting tubes), that is,

Emerged brood cells=total number of brood cells∗(%parasite free‐%non-emerged)

where total number of brood cells is the average number of eggs laid in individual nesting tubes, %parasite‐free is the average proportion of brood cells that were not attacked by parasites, and %non-emerged is the average proportion of non‐emerged brood cells for reasons other than parasites.

Offspring production is calculated using three different reproductive traits that are likely to show trade‐offs as part of different reproductive strategies aiming at maximizing individual reproduction success. Such strategies are expected to be underpinned by similar mechanisms across species and taxa that share the same reproductive system, such as nesting in cavities. Therefore, both offspring production and the traits responsible are likely to be comparable between cavity‐nesting bees and wasps.

Building upon the previous work above, our study aims to (a) determine whether and how reproductive traits, trait diversity, and offspring production respond to multiple environmental variables underlying an urbanization gradient and (b) investigate how well reproductive trait variation explains individual offspring production.

Cavity‐nesting bee and wasp reproductive traits (Box 1) should respond to local variation in resource availability and pathogen and parasite communities across urbanization gradients. Moreover, these traits are involved in multiple trade‐offs. In an ideal scenario, individual mothers would aim to maximize the total number of brood cells and minimize offspring mortality (due to parasites or other factors). However, behaviors that increase foraging time simultaneously increase both brood provisioning and parasite exposure, and dividing provisions across many offspring may decrease the survival probability of each (Zurbuchen et al., 2010). Because of these trade‐offs, there may be several different but equally successful phenotypes. Investigating these phenotypes can reveal the distribution of reproductive strategies within a community and the consequences for overall or individual offspring production (e.g., Pistón et al., 2019).

Reproductive traits have been shown to be sensitive to environmental conditions (e.g., Sedinger et al., 1995; Stenseth & Mysterud, 2002) and therefore should provide a direct mechanism by which population growth and persistence varies across urbanization gradients. To better understand how different species respond to environmental conditions, it is crucial to consider multiple traits simultaneously (i.e., trait combinations or multidimensional phenotypes), as well as both interspecific and intraspecific trait covariation and their link to fitness (Laughlin & Messier, 2015). In this context, selective pressures such as environmental gradients can lead to the convergence of phenotypes toward an optimum (e.g., Laughlin & Messier, 2015; MacArthur & Levins, 1967). However, multiple optima (i.e., divergent phenotypes with comparable fitness) are also possible and have been detected in both plants (e.g., Pistón et al., 2019) and animals (e.g., Calsbeek et al., 2010). The coexistence of multiple, equally fit phenotypes is often the result of competition for limiting resources (MacArthur & Levins, 1967), which leads to increased niche partitioning. This has been observed in other insects when analyzing intraspecific variation in reproductive strategies (Chao et al., 2013).

Convergence or divergence across multiple traits can be detected using appropriate metrics of trait distribution. Therefore, we quantified how our measures per nesting tube were distributed within communities and how our three reproductive traits influenced offspring production and responded to environmental variables by calculating three complementary trait diversity measures: *trait richness* (the amount of trait space occupied by the community), *trait evenness* (the regularity in the distribution of the trait values within the community, as a measure of niche partitioning), and *trait divergence* (the degree to which trait values are spread around the community average phenotype) (Mason et al., 2005). By capturing different aspects of trait diversity, these indices probe the mechanistic effects of urbanization on reproductive success. In this respect, we predict that different trait diversity measures will affect offspring production as consequences of selective processes driven by environmental conditions and biotic interactions.

In general, if food and nesting resources decrease with urbanization, we expect total offspring production to also decline in urban areas. We predict increased rates of brood failure due to parasitism and pathogens with urbanization if bees and wasps must increase their foraging time to access preferred resources. In terms of trait diversity, we first hypothesize that trait richness and trait divergence will decline with increasing urbanization due to convergence toward fewer, well‐adapted phenotypes. Second, we hypothesize that trait evenness may increase if the reproductive traits involved also reflect resource acquisition strategies (see Box 1), and therefore competition for resources in densely urban areas imposes selection for a more uniform distribution within the viable trait space (Fontana et al., 2016; Mouchet et al., 2010). That is, we may expect greater trait evenness (increased regularity in the distribution of reproductive traits) in heavily urbanized sites if competition for specific resources requires more frequent or longer foraging trips for some organisms, driving limiting similarity, and niche partitioning (e.g., MacArthur & Levins, 1967). Consequently, because bees and wasps should perform best in communities where resources are more evenly partitioned and competition is reduced, our third and final hypothesis was that offspring production will increase with trait evenness, independent of trait richness and divergence.

## 2 METHODS

### 2.1 Study area and sampling design

We sampled cavity‐nesting solitary bees and wasps over 3 years (2011–2013), in the city of Toronto, Ontario, Canada (approx. 630‐km^2^ and 2.8 million inhabitants). Our study occurred in a single city and therefore may not be generalizable to other cities. However, Toronto is Canada's largest city and contains a broad gradient of urbanization comparable in area and dimension to other major cities across North America. We used trap nests, which provide nesting habitat for these insects (Staab et al., 2018). Trap nests were set up from May until October, covering the whole nesting season of both bees and wasps. Each trap nest consisted of a piece of PVC pipe 30‐cm long and 10‐cm in diameter, with thirty 15‐cm long cardboard nesting tubes (ten of three different widths each: 3.4, 5.5, 7.6 mm) and a pipe cap to close the opposite end of the trap. Each trap nest was attached to a fixed feature at each site to ensure the trap nest entrance was unobscured and in full to partial sun. We set up 200 trap nests (1 per site), 153 of which were (a) set up during at least 2 out of 3 years, (b) colonized in at least 1 year, and (c) located within the City of Toronto boundary. These sites covered four distinct types of urban green space types: *community gardens* (*n* = 15), where community cultivated crops and other plants in public spaces, *home gardens* (*n* = 70), owned and maintained by individuals and occurring behind their properties and surrounded by fencing, *urban parks* (*n* = 48), variable in size and maintained by city staff, and *green roofs* (*n* = 20), on top of public and private buildings. All sites were >250‐m apart and no spatial autocorrelation was detected in bee community composition among all sites surveyed (MacIvor & Packer, 2015).

### 2.2 Environmental variables

**FIGURE A1:**
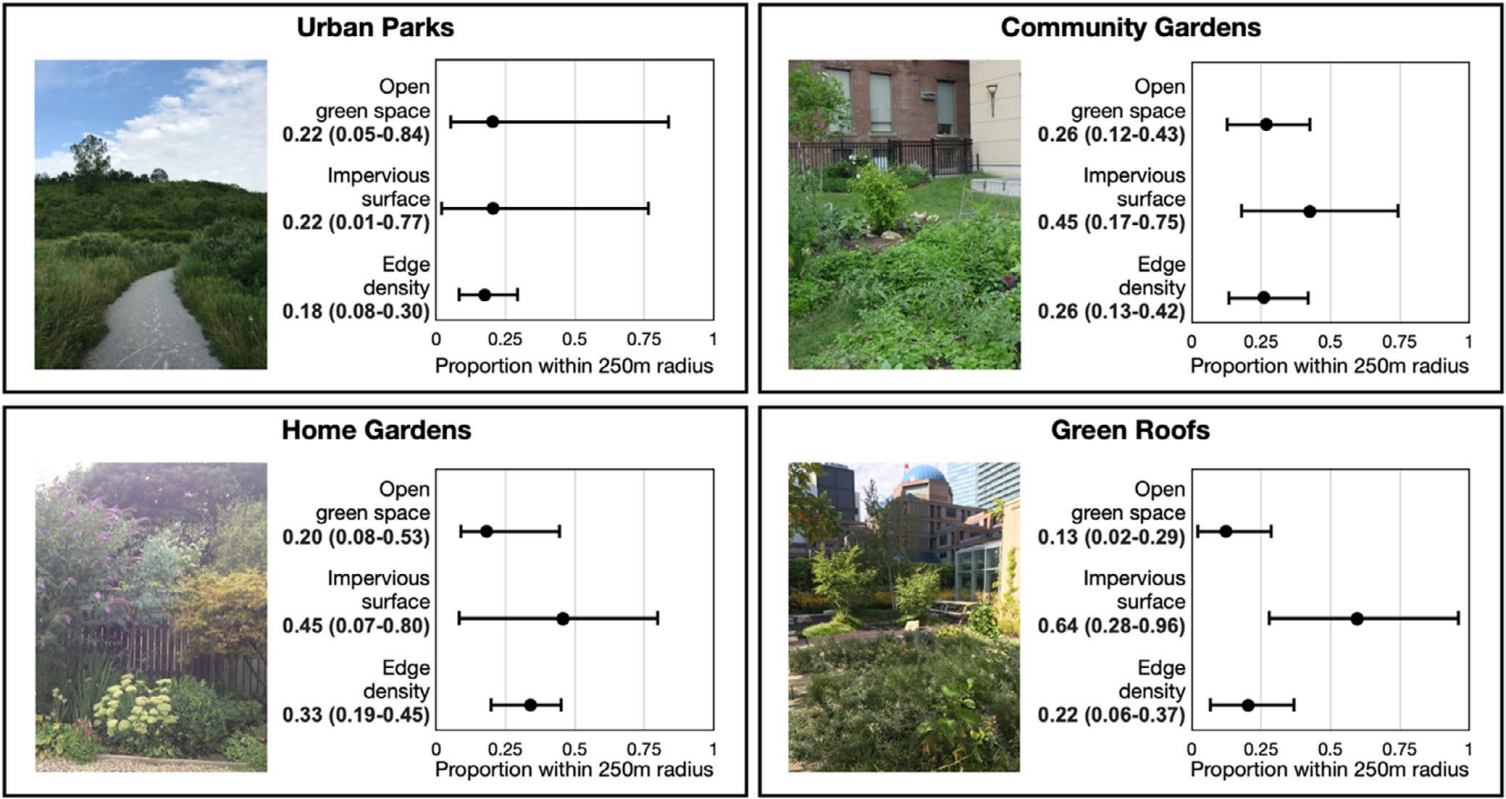
Description of the urban green space (UGS) types in the study. Barplots show the mean of proportion and range (min–max) of each environmental variable within 250 m radius for each of the UGS types used in this study. Urban parks contained the greatest variation in “open green space,” and the lowest “impervious surface,” home gardens contained the greatest “edge density” and green roofs the highest impervious surface. Otherwise, all UGS types comprised overall similar urban characteristics

For each site, we estimated the proportion of eight landscape cover classes (forested area, open green area, bare soil, water, buildings, roads, other paved surfaces, and agriculture) at a 250‐m radius using the 2007 Forest and Land Cover dataset (0.6‐m raster pixel resolution) (City of Toronto, 2009) and the *spatialEco* package (Evans & Ram, 2021) in R (R Development Core Team, 2018). From these landscape cover classes, we calculated three environmental variables: (a) open green area, (b) impervious surface (the sum of the proportion of buildings, roads, and other paved surfaces), and (c) edge density, a measure of heterogeneity, as the sum of the lengths (m) of all edge segments of all landscape cover classes for each site calculated using the *SDMtools* package (VanDerWal et al., 2014) and divided by total area (m^2^) (Figure A1). These three environmental variables provide complementary information about the availability of habitat and were not correlated (maximum pairwise Pearson's *r* = 0.4), so we retained all three for subsequent analyses. We expect that variation in these urbanization gradients, and in green space types, across the city will capture variation in the type and availability of food and nesting resources important for bees and wasps, as well as the prevalence of pathogens and parasites.

### 2.3 Notes on trait selection and measurement

Cavity‐nesting bees and wasps provision brood cells in a linear series from the back of the nesting tube to the front, such that a single tube contains multiple offspring from an individual female (MacIvor, 2017). Single nests are commonly built by one single female (Danforth et al., 2019), although two different species were observed in a single nest on occasion, for example, if one usurps the nest from another. The number of nests a single female can build in a season, and how eggs are distributed among nesting tubes, are not known. For this reason, we refer to the reproductive output of single nesting tubes, rather than of a single maternal individual (Sedinger et al., 1995), irrespective whether they reflect the entire brood of a female or only part of it. For these reasons, and because reproduction is one portion of the life cycle, our reproductive traits are not intended to quantify lifetime fitness.

We also note here that variation in reproductive strategies can happen at both the interspecific and intraspecific level, and we combine both sources of variation in our analyses. We are aware that other important traits related to offspring production and survival, such as sex ratio, paternal care, and nest defense (e.g., in wasps: *Trypoxylon*, in bees: *Megachile pugnata*; Krombein, 1967) are potentially relevant, but it was not possible to quantify them in our study. However, we believe the traits we used are better suited to the investigation of reproductive success of individual nests compared with traits typically used for bees and wasps, such as morphological features of species related to mobility, resource use (foraging and nesting), and phenology.

### 2.4 Bee and wasp diversity

Each season in October, trap nests were retrieved, all nesting tubes were opened, and each individual brood cell removed. Individuals were stored in 24‐well assay trays at 4°C until April and then moved to a growth chamber at 26°C and ~65% humidity. Over the following weeks, all cavity‐nesting bees, wasps, and their parasites emerged, then were identified to species using synoptic collections at York University and the University of Guelph, dichotomous keys (*Megachile*: (Sheffield et al., 2011); other bees: (Ascher & Pickering, 2016; Mitchell, 1960, 1962); all vespid wasps: (Buck et al., 2008), and DNA barcoding services provided by the Centre for Biodiversity Genomics at the University of Guelph. All completed brood cells containing non‐emerged juveniles per nesting tube were also counted to estimate the number of nonparasitized offspring that did not emerge due to inadequate food supply, fungal or bacterial infection, or some other factor. All specimens are curated in the BUGS lab collections at the University of Toronto Scarborough.

### 2.5 Functional indices

Using the three reproductive traits (Box 1), we calculated trait *richness* (TOP), trait *evenness* (TED), and trait *divergence* (FDis, see Introduction). TOP and TED were explicitly developed for the use of individual‐based traits and therefore are sensitive to changes in the position of single data points within the trait space (see Fontana et al., 2016). TOP quantifies the occupied trait space by considering the arrangement of all individuals within concentric layers and decreases when the environment selects for a reduced number of viable trait combinations. TED measures the regularity in the distribution of individuals within the trait space by comparing it with a perfectly even reference distribution and can respond to competitive interactions (Fontana et al., 2016). FDis is calculated as the mean distance of individuals from the center of gravity of their distribution in multiple dimensions; FDis increases, for example, when extreme phenotypes become more prevalent (Laliberté & Legendre, 2010). Prior to index calculation, we checked pairwise correlations between traits across all nesting tubes in our full dataset. Correlations were low (maximum Pearson's *r* = 0.31), so all traits were retained. We z‐transformed traits so they would carry equal weight in our trait diversity measures (Petchey & Gaston, 2006).

### 2.6 Analyses

The analyses were carried out in two consecutive steps. First, since trap nests varied in the number of occupied nesting tubes, trait diversity metrics (TOP, TED, FDis) and mean values of the three reproductive traits were calculated by performing a bootstrap procedure to randomly select seven nesting tubes from each site 999 times. To do that, we only considered those sites with at least eight occupied nesting tubes, and for bees and wasps separately, leaving us with 137 nesting sites in the final analyses (*N* = 77 for bees and *N* = 115 for wasps; UGS types: community garden *N* = 8 and 9, home garden *N* = 35 and 56, park *N* = 27 and 41, green roof *N* = 7 and 9, respectively). All data on total brood cells, parasitism, and failure for each nest evaluated in the final analyses are included in the Appendices S1‐S3 accompanying the manuscript. We chose seven as the number of tubes to randomly select, since this yielded reliable trait diversity estimates by allowing our nesting tube sample size to exceed the number of traits considered, without having to eliminate too many trap nest sites due to low occupancy (thus maintaining statistical power). By focusing on trap nest sites with at least eight occupied nesting tubes, we ensured that bootstrapping would preserve enough variability in trait diversity estimates in all sites considered. Next, we averaged all bootstrapped trait diversity and mean reproductive trait values for further analyses.

Upon checking model assumptions visually, we decided to consistently use linear models throughout our analyses. For each combination of predictors and response variables, we fitted four models: (a) a simple linear model, (b) a model including UGS type and its interaction with the predictor, to assess whether UGS type affects slope estimates, (c) a model including a quadratic term for the predictor, thus allowing a parabolic relationship, and (d) a model including both the interaction with UGS type and the quadratic term. We then defined the best model as the one with the smallest AICc. This best univariate model determined whether a given predictor will include an interaction with UGS type and/or a quadratic term when fitting our multivariate models, in which bees and wasps were always modeled separately. First, to determine how bee and wasp trait structure changes across an urbanization gradient, we fit several multivariate models with all three urban environmental variables (open green area, impervious surface, and edge density) as predictors of distinct response variables: community trait diversity (TOP, TED and FDis), mean trait values (across all bees or wasps within a site), and the offspring production measured as the mean number of emerged brood cells of bees or wasps at each nesting site. Second, we modeled offspring production as a function of the three trait diversity metrics (TOP, TED, FDis) to test the hypothesis that offspring production is affected by trait diversity, which is the consequence of selective processes driven by environmental conditions and biotic interactions. Please note that the trait diversity metrics used here describe the distribution of the three reproductive traits in the community functional space, which is fully independent from their mean values used for calculating the offspring production. Therefore, the relationships between trait diversity metrics and offspring production will not result from trivial correlations.

## 3 RESULTS

From the trap nests examined, we identified 31 species of cavity‐nesting bee in eight genera and three families (Table 1). Home gardens had the most bee species and highest proportion of nests provisioned across UGS types (Table A1). Green roofs were unique among sites analyzed because they were dominated by a single bee species, *Megachile rotundata* (62.0% of all bee nests from green roofs). On average, only 6.8% of bee brood cells were parasitized, and 25.7% suffered mortality due to other environmental factors.

**TABLE 1:** Summary of offspring production and mortality factors from nests of cavity‐nesting bees and wasps evaluated in this study

	Bees (*N* = 77 sites)	Wasps (*N* = 115 sites)
Total nests evaluated	1,536	2,537
Average (min–max) nests per site	19.95 (8–64)	22.06 (8–51)
Average (min–max) brood cells per nest	6.68 (1–24)	4.16 (1–20)
Average (min–max) % parasite‐free cells	93.2 (0–100)	90.2 (0–100)
Average (min–max) % non‐emerged cells	25.7 (0–100)	30.7 (0–100)
Average (min–max) emerged brood cells per nest	5.20 (0–24)	2.89 (0–19)
Most abundant genera	*Osmia* (46.9% of bee genera)	*Trypoxylon* (52.3% of wasp genera)
Most abundant species	*Osmia caerulescens* (22.8%) *Osmia pumila* (22.4%) *Megachile rotundata* (15.7%)	*Trypoxylon frigidum* (25.9%) *Trypoxylon collinum* (20.0%) *Psenulus pallipes* (8.4%)

We identified 20 species of cavity‐nesting wasp from ten genera and four families (Table 1). Among UGS types analyzed, parks had the highest number of wasp species and nests provisioned (followed by home gardens), whereas green roofs had the fewest species and least nests provisioned (Table A2). Compared with bees, wasps suffered greater rates of parasitism (9.8%) and mortality from other factors (30.7%) (Table 1).

**FIGURE 1:**
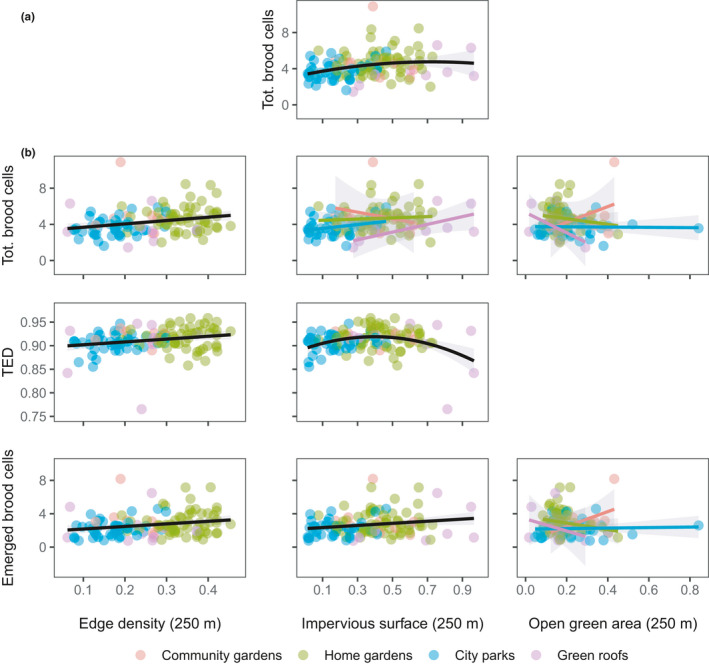
Plots of all significant univariate best models with environmental variables as predictors for bees (a) and wasps (b). Model fits include gray 95% confidence intervals (sometimes barely visible). For bees, the only significant model (impervious surface vs. total brood cells) included a quadratic term. This was also the case for a wasp model (impervious surface vs. TED), while three other wasp models included an interaction with UGS type (colored regression lines). More details are reported in Tables A3 and A4

Environmental variables were generally poor predictors of trait diversity, offspring production, and mean values of single reproductive traits (Table 2, Figure 1, Tables A3 and A4). For bees, none of the multivariate models with environmental variables as predictors were statistically significant (Table 2), and only one univariate model was significant (Table A3): the total number of bee brood cells peaked at intermediate levels of impervious surface, but the curvature of the parabola was weak (Figure 1).

**TABLE 2:** Multivariate full models of bees and wasps, relating environmental gradients within 250‐m (Open green area + Impervious surface + Edge density) to single traits (Proportion non‐emerged cells, Proportion parasite‐free cells, and Total brood cells), trait diversity indices (TOP = trait onion peeling, TED = trait evenness distribution, FDis = functional dispersion) and Emerged brood cells (offspring production), and trait diversity indices to Emerged brood cells

Predictors	Response variable	*R* ^2^	*p*‐value
Bees			
Environmental gradients	Prop. non‐emerged cells	0.02	0.631
	Prop. parasite‐free cells	0.04	0.348
	Total brood cells	0.11	0.085
	TOP	0.04	0.525
	TED	<0.01	0.919
	FDis	0.02	0.676
	Emerged brood cells	0.09	0.162
Trait diversity indices	Emerged brood cells	0.43	<0.001***
Wasps			
Environmental gradients	Prop. non‐emerged cells	0.01	0.643
	Prop. parasite‐free cells	0.01	0.703
	Total brood cells	0.23	0.005**
	TOP	0.01	0.749
	TED	0.12	0.012*
	FDis	0.03	0.399
	Emerged brood cells	0.16	0.028*
Trait diversity indices	Emerged brood cells	0.45	<0.001***

Note

Single predictors include an interaction with UGS type, a quadratic term or both, when the corresponding univariate models had the smallest AICc (Tables A3 and A4).

* *p*<0.05 (0.01<*p*<0.05)

** *p*<0.01 (0.001<*p*<0.01)

*** *p*<0.001

In contrast, some measures of wasp trait structure showed a significant relationship with environmental variables in multivariate models (Table 2): total brood cells (*R* ^2^ = 0.23), trait evenness (TED; *R* ^2^ = 0.12), and emerged brood cells (*R* ^2^ = 0.16). Univariate models (Figure 1 and Table A4) show a weak increase in trait evenness (TED), total brood cells, and emerged brood cells with increasing landscape heterogeneity (edge density). Along the impervious surface gradient, wasp trait evenness (TED) peaked at intermediate levels (Figure 1), although this unimodal trend appears heavily influenced by a few less diverse and highly impervious green roof sites. The number of emerged brood cells per nest in wasps slightly increased with impervious surface, while the relationship between impervious surface and the total number of brood cells depended on UGS type, as shown by the interaction included in the selected model (Figure 1). Single‐UGS type relationships were all positive, except for community gardens (negative), with the strongest increase in total number of brood cells with increasing impervious area shown by green roofs (Figure 1). The relationships between open green area and emerged brood cells, as well as total brood cells, also varied with UGS type in wasps (Figure 1).

**FIGURE 2:**
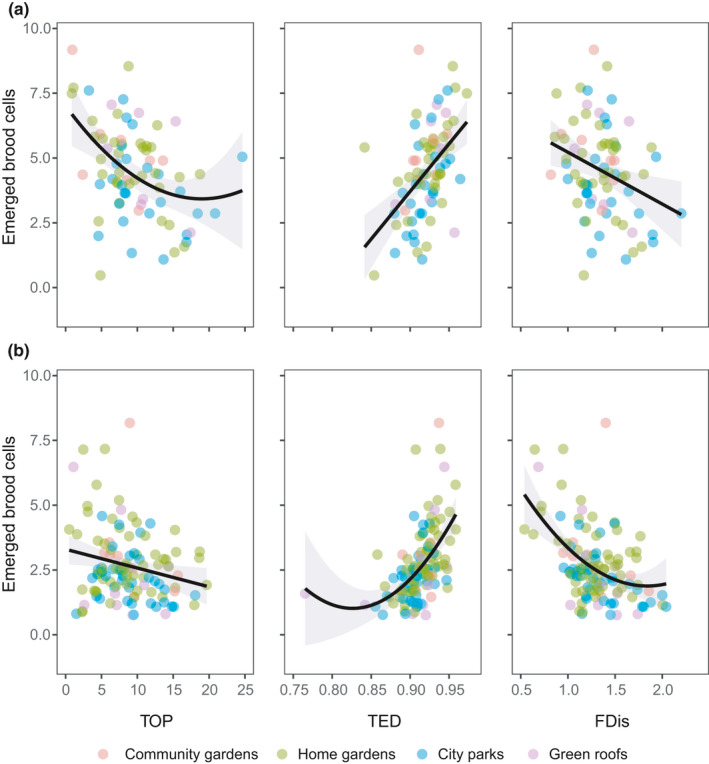
Relationships between trait diversity indices (TOP, TED, and FDis) and offspring production (mean number of emerged brood cells) for bees (a) and wasps (b). These variables were characterized at single trap nest sites (*N* = 77 for bees, *N* = 115 for wasps) by bootstrapping (999 times) seven individual nesting tubes. All linear model fits were significant (black lines with gray 95% confidence intervals). Statistical values are shown in Tables A3 and A4

The offspring production (i.e., mean number of emerged brood cells per site) was significantly related to trait diversity indices: the multivariate model had an *R* ^2^ of 0.43 in bees and 0.45 in wasps (Table 2), while in univariate models the relationships were similar across both taxa, with *R* ^2^ values ranging from 0.05 to 0.29 (Figure 2, Tables A3 and A4). When a quadratic term was included in the best model (TOP for bees, TED, and FDis for wasps), only about half of the parabolic curve was present and the region of maximum curvature (including the minimum) was associated with high uncertainty (as indicated by large confidence intervals in Figure 2). In synthesis, for both bees and wasps, TOP and FDis showed a negative relationship with the number of emerged brood cells, while TED had a positive influence (Figure 2).

## 4 DISCUSSION

Our study assessed how reproductive traits and trait diversity of cavity‐nesting solitary bee and wasp communities responded to multiple urban environmental variables. We evaluated whether community variation in reproductive traits explained individual offspring production to assess whether urbanization could impact population persistence by constraining trait diversity. We build upon the growing urban biodiversity literature in three ways. First, we evaluate functional traits, which allow us to understand biodiversity patterns based on function rather than species identity (McGill, Enquist, Weiher, & Westoby, 2006). Second, we link reproductive traits to environmental conditions, which has been shown to be important in structuring community‐level patterns (e.g., Bolnick et al., 2011; Cadotte, Carscadden & Mirotchnick, 2011). Third, we quantified reproductive output. By doing this, we gained insight into the possible mechanistic links between environment, community composition, and ultimately individual reproductive output that could be tested in additional cities.

### 4.1 Trait diversity affects offspring production

We hypothesized that reproductive trait diversity in cavity‐nesting bees and wasps would reflect the mechanisms that drive offspring production across urban environmental variables. We note that reproductive traits were measured at the individual level (by focusing on single nesting tubes), but offspring production was then averaged across nesting tubes (see Box 1) to model its responses to community‐level trait diversity. With intraspecific trait variation, we included an important driver of population‐ and community‐level processes (e.g., Bolnick et al., 2011). We observed a consistent pattern in bees and wasps: offspring production was positively correlated with trait evenness (TED) and negatively with trait richness (TOP) and trait divergence (FDis) (Figure 2).

We interpret the observed contrasting patterns as reflecting different strategies for optimizing the reproductive success of the individual nesting tubes given trade‐offs between reproductive traits. In our study, reproductive trait diversity (especially in wasps) is partially explained by environmental variables, whereas trait diversity in bees did not respond significantly to the environmental variables investigated. The reduction in wasp trait divergence (FDis) in densely urbanized areas suggests individuals converge upon a narrow suite of reproductive trait combinations in areas characterized by impervious infrastructure. Independent of the responses to environmental variables, the observed negative relationships between trait diversity (richness and divergence) and offpring production suggests the existence of an optimal reproductive strategy that maintains consistent reproductive success of bees and wasps across the urban landscape. Optimal trait combinations are known to be context dependent and dynamic (Everaars et al., 2018; Parker & Smith, 1990); with urbanization, bees and wasps must continually balance access to forage and nesting materials with access to ideal nesting locations, and foraging time with nest defense against parasites and competitors for nesting space.

We speculate that a reduction in reproductive trait diversity could, in turn, lead to possible competition among individual bees and wasps sharing similar foraging and nesting strategies. Therefore, within this reduced available trait space (low trait richness and divergence), regular functional spacing among individual bees and wasps (high trait evenness) is expected to minimize possible competition for nesting opportunities, leading to a higher reproductive success (more emerged brood cells), as observed in our study. To our knowledge, there is little other evidence at the community level that anthropogenic change generally leads to higher trait evenness in a reduced functional space. Recent studies show that similar patterns exist along other environmental variables in an aquatic system; e.g., phytoplankton communities showed increasing evenness of traits related to photosynthesis when exposed to reduced light (Fontana et al., 2018; Fontana et al., 2019). Similarly, reproductive traits in pine trees became more evenly distributed under severe drought conditions (He et al., 2018). The relationship between reproductive trait diversity metrics and reproductive success of cavity‐nesting bees and wasps is consistent across UGS types in our study, suggesting a possible common mechanism that deserves future investigation.

### 4.2 Weak trait response to environmental variables

We found no evidence that reproductive traits, trait diversity, or offspring production in bee communities responded to our three environmental variables (Table 2). In wasps, only the total number of brood cells, reproductive trait evenness, and offspring production were slightly affected by urbanization (Table 2). We note that, as we did not correct for multiple testing, these trait responses might be the result of a Type I error. As a consequence, we conclude that the reproductive traits and trait diversity of cavity‐nesting bees and wasps did not decline (or declined only marginally) with increasing urbanization level. A similar pattern, though using different traits, was observed by Bartomeus et al. (2018) in crop pollination systems along gradients of land‐use change. The absence of strong trends in reproductive traits and trait diversity with increasing urbanization levels suggests that previously reported declines in bee species richness and abundance in densely urban areas (Fortel et al., 2014) may not translate to homogenization of reproductive traits, at least within cavity‐nesting bee and wasp communities that use trap nests. The maintenance of functional diversity related to reproductive strategies despite increasing urbanization might indicate that environmental variables in cities create a spatial mosaic of available resources. For example, two mason bees in our study forage on white clover (*Trifolium repens* L.) (MacIvor et al., 2014), which is widespread in Toronto (and other cities), and generalist *Trypoxylon* wasps consume abundant spider taxa (Coville, 1981). For wasps, changes in reproductive trait diversity might be more affected by prey habitat requirements and likely to not decrease with urbanization (e.g., *Passaloecus* and *Psenulus* wasps collect aphids, which are common pests of many cultivated plants in cities). Fine‐scale food availability could be consistent across an urbanization gradient but difficult to detect using coarser land cover data. This could also explain why we did not find any differences in offspring production along our environmental variables. In sum, if food or nesting resources (or other unmeasured environmental variables) are a major determinant of bee and wasp reproductive traits and trait diversity but are patchy and occur at small spatial scales, relatively independent of the overarching urbanization gradient, and not specific to particular UGS types, we may not observe the expected reduction in offspring production and biodiversity in densely built areas.

## 5 LIMITATIONS AND NEXT STEPS

The factors that we did not measure explicitly in this study, such as dispersal, mortality, interspecific competition, predation, and habitat destruction (Sattler et al., 2010) may also affect reproductive trait diversity in our system. Cities represent a dynamic system in which bee and wasp communities may not be in equilibrium. Time lag effects may also contribute to the lack of functional responses to classical environmental variables sampled at a given point in time and space. Longitudinal studies may be invaluable in teasing apart some of our suggested mechanisms here, to reveal whether spatiotemporal composition and configuration of particular UGS within the urban matrix (Colding, 2007) drive species assemblages and distribution, as well as reproduction, growth, and survival of single individuals. Although our consideration of different UGS types represents a step forward in treating the city as a habitat mosaic, future studies that characterize the spatial configuration of UGS types would be able to test whether adjacent contrasting UGS types increase biodiversity by providing complementary habitat and resources. Similarly, fine‐scale floral and habitat resources may be more direct drivers of bee and wasp communities, compared with coarser gradients of environmental heterogeneity, impervious surface, and greenness in cities. Therefore, higher resolution mapping of floral and habitat resources (e.g., Libran‐Embid et al., 2020) might further reveal how local factors influence these organisms in urban landscapes (Williams & Winfree, 2013). Lastly, additional trap nest studies in other cities would reveal whether similar trends emerge and suggest other landscape characteristics that may influence communities of cavity‐nesting bees and wasps.

## CONFLICT OF INTEREST

This article does not present research with ethical considerations.

## AUTHOR CONTRIBUTIONS

**Marco Moretti:** Conceptualization (equal); Methodology (equal); Writing‐original draft (lead); Writing‐review & editing (lead). **Simone Fontana:** Conceptualization (equal); Data curation (equal); Formal analysis (lead); Methodology (equal); Writing‐review & editing (equal). **Kelly A. Carscadden:** Conceptualization (equal); Methodology (equal); Writing‐review & editing (equal). **J. Scott MacIvor:** Conceptualization (equal); Data curation (lead); Funding acquisition (lead); Investigation (lead); Methodology (equal); Supervision (equal); Writing‐review & editing (equal).

**TABLE A1:** Each identified bee species and the number of nests used in the analysis (*N* = 77 sites) per urban green space type (UGS)

Family	Species	UGS types			
		Community (*N* = 8)	Green roof (*N* = 7)	Home (*N* = 35)	Park (*N* = 27)
Apidae	*Anthophora terminalis* Cresson				1
Megachilidae	*Anthidium manicatum* (Linnaeus, 1758)	2	1		2
	*Megachile brevis* Say, 1837				4
	*Megachile campanulae* (Robertson, 1903)	10	6	133	39
	*Megachile centuncularis* (Linnaeus, 1758)	4	10	45	97
	*Megachile frigida* Smith, 1853			8	2
	*Megachile inermis* Provancher, 1888			4	
	*Megachile mendica* Cresson, 1878			4	1
	*Megachile pugnata* Say, 1837	5		2	39
	*Megachile relativa* Cresson, 1878	2		2	2
	*Megachile rotundata* Fabricius, 1787	13	105	62	58
	*Megachile sculpturalis* Smith, 1853			4	
	*Heriades carinata* Cresson, 1864	18	9	65	23
	*Heriades variolosa* (Cresson, 1872)			1	
	*Chelostoma campanularum* (Kirby, 1802)			4	
	*Chelostoma rapunculi* Lepeletier, 1841	1		2	
	*Hoplitis producta* (Cresson, 1864)	1			2
	*Hoplitis spoliata* (Provancher, 1888)			4	9
	*Hoplitis truncata* (Cresson, 1878)			1	
	*Osmia atriventris* Cresson, 1864	1		3	
	*Osmia caerulescens* (Linnaeus, 1758)	90	29	132	123
	*Osmia lignaria* Say, 1837	6	1	9	7
	*Osmia pumila* Cresson, 1864	12	2	157	102
Colletidae	*Hylaeus affinis* Smith, 1853	1		5	
	*Hylaeus annulatus* (Linnaeus, 1758)	1			7
	*Hylaeus hyalinatus* Smith, 1842	10			
	*Hylaeus leptocephalus* Morawitz, 1871	1			
	*Hylaeus mesillae* (Cockerell, 1896)			5	
	*Hylaeus modestus* Say, 1837	2		13	5
	*Hylaeus verticalis* (Cresson, 1869)				1
	*Hylaeus sp*.		3	1	
	Total nests provisioned	180	166	666	524

**TABLE A2:** Each identified wasp species and the number of nests used in the analysis (*N* = 115 sites) per urban green space type (UGS)

Family	Species	UGS types			
		Community (*N* = 9)	Green roof (*N* = 9)	Home (*N* = 56)	Park (*N* = 41)
Crabronidae	*Passaloecus cuspidatus* Smith, 1856	11		19	6
	*Passaloecus gracilis* (Curtis, 1834)	5	5	59	30
	*Passaloecus monilicornis* Dahlbom, 1842	1			
	*Psenulus pallipes* (Panzer, 1798)	17	20	57	31
	*Trypoxylon collinum* Smith, 1856	34	62	202	157
	*Trypoxylon frigidum* Smith, 1856	51	8	432	89
	*Trypoxylon lactitarse* Saussure, 1867	6	5	24	144
Pompilidae	*Auplopus mellipes* (Say, 1836)	13		13	45
	*Dipogon sayi* Banks, 1941				10
Sphecidae	*Isodontia mexicana* (Saussure, 1867)	11	35	157	113
	*Monobia quadridens* (Linnaeus, 1763)	5		4	5
Vespidae	*Ancistrocerus adiabatus* (Saussure, 1852)			6	16
	*Ancistrocerus antilope* (Panzer, 1798)	3		16	80
	*Ancistrocerus gazella* (Panzer, 1798)		1		2
	*Euodynerus foraminatus* (Saussure, 1853)	4	1	15	65
	*Euodynerus planitarsis* (Bohart, 1945)				7
	*Symmorphus albomarginatus* (Saussure, 1855)				2
	*Symmorphus bifasciatus* (Linnaeus, 1761)				1
	*Symmorphus canadensis* (Saussure, 1855)	7		47	167
	*Symmorphus cristatus* (Saussure, 1855)	45		23	143
	Total nests provisioned	213	137	1,074	1,113

**TABLE A3:** Bee univariate models relating environmental variables to single traits, trait diversity and reproductive output (A), and trait diversity to reproductive output (B)

Predictor	Response variable	AICc	AICc interaction UGS type	AICc quadratic term	AICc interaction + quadratic term	*R* ^2^	*p*‐Value	
(A)							
Edge density	Prop. non‐emerged cells	**−74.7**	−66.4	−73.1	−58.6	<0.01	0.769	
	Prop. parasite‐free cells	−**169.7**	−159.5	−168.2	−152.5	0.02	0.185	
	Total brood cells	**302.3**	304.4	303.5	309.0	0.02	0.196	
	TOP	**461.1**	469.2	462.0	479.0	0.03	0.164	
	TED	−**349.9**	−337.8	−347.7	−330.3	<0.01	0.611	
	FDis	**22.4**	31.3	22.5	41.1	0.02	0.235	
	Emerged brood cells	**312.4**	315.0	313.2	320.7	0.02	0.209	
Impervious area	Prop. non‐emerged cells	**−74.8**	−72.7	−73.2	−63.2	<0.01	0.663	
	Prop. parasite‐free cells	**−168.3**	−158.1	−168.2	−153.5	<0.01	0.500	
	Total brood cells	302.5	306.3	**298.8**	314.9	0.09	0.028*	
	TOP	463.1	469.7	**462.3**	468.6	0.04	0.241	
	TED	**−349.6**	−341.7	−347.7	−331.1	<0.01	0.824	
	FDis	**23.2**	31.7	24.6	35.7	<0.01	0.425	
	Emerged brood cells	313.4	316.4	**310.8**	324.8	0.07	0.072	
Open green space	Prop. non‐emerged cells	**−75.9**	−65.7	−73.7	−69.1	0.02	0.265	
	Prop. parasite‐free cells	**−168.6**	−159.5	−166.4	−149.6	<0.01	0.406	
	Total brood cells	**303.0**	311.3	303.6	316.2	0.01	0.318	
	TOP	**463.0**	471.3	464.6	480.6	<0.01	0.844	
	TED	**−350.0**	−338.3	−347.8	−328.6	<0.01	0.551	
	FDis	**23.8**	33.1	26.0	41.7	<0.01	0.869	
	Emerged brood cells	**312.5**	320.9	314.1	325.2	0.02	0.217	
(B)							
TOP	Emerged brood cells	299.5	309.4	**298.9**	316.0	0.20	<0.001***	
TED		**292.5**	300.0	292.9	300.1	0.24	<0.001***	
FDis		**306.6**	317.3	308.7	325.9	0.09	0.007**	

Note

For each combination of predictor and response variable, we compared a simple linear model with models including UGS type and its interaction with the predictor, a quadratic term for the predictor, and both the interaction and the quadratic term. We defined as the best model the one with the smallest AICc (bold). *R* ^2^ and *p*‐values are only indicated for the best model.

* *p*<0.05 (0.01<*p*<0.05)

** *p*<0.01 (0.01<*p*<0.01)

*** *p*<0.01

**TABLE A4:** Wasp univariate models relating environmental variables to single traits, trait diversity, and reproductive output (A), and trait diversity to reproductive output (B)

Predictor	Response variable	AICc	AICc interaction UGS type	AICc quadratic term	AICc interaction + quadratic term	*R* ^2^	*p*‐Value	
(A)							
Edge density	Prop. non‐emerged cells	**−95.6**	−85.8	−93.5	−77.5	<0.01	0.438	
	Prop. parasite‐free cells	**−218.7**	−210.4	−216.6	−202.7	<0.01	0.391	
	Total brood cells	**407.4**	409.2	409.6	413.8	0.07	0.005**	
	TOP	**664.1**	673.4	666.2	681.1	<0.01	0.624	
	TED	**−516.2**	−507.0	−514.5	−498.0	0.05	0.012*	
	FDis	**47.4**	55.2	49.6	62.1	0.02	0.120	
	Emerged brood cells	**402.6**	405.6	404.8	412.0	0.05	0.017*	
Impervious area	Prop. non‐emerged cells	**−95.1**	−84.1	−94.8	−75.6	<0.01	0.705	
	Prop. parasite‐free cells	**−219.0**	−208.5	−217.1	−204.3	<0.01	0.322	
	Total brood cells	407.9	**404.9**	408.6	408.2	0.19	0.002**	
	TOP	**664.3**	675.8	665.9	680.8	<0.01	0.992	
	TED	−509.7	−512.6	**−522.6**	−504.7	0.12	<0.001***	
	FDis	**49.6**	60.6	51.7	64.1	<0.01	0.577	
	Emerged brood cells	**404.1**	408.4	405.3	415.5	0.04	0.040*	
Open green space	Prop. non‐emerged cells	**−95.4**	−86.0	−93.3	−76.8	<0.01	0.544	
	Prop. parasite‐free cells	**−218.4**	−208.3	−216.4	−203.1	<0.01	0.504	
	Total brood cells	415.3	**404.5**	417.3	407.2	0.19	0.002**	
	TOP	**663.3**	673.9	665.4	679.2	<0.01	0.326	
	TED	−510.9	−509.5	**−512.1**	−502.4	0.04	0.108	
	FDis	**49.1**	59.7	51.0	61.8	<0.01	0.369	
	Emerged brood cells	408.4	**404.5**	410.5	411.3	0.14	0.020*	
(B)							
TOP	Emerged brood cells	**402.7**	404.7	404.8	409.1	0.05	0.018*	
TED		378.9	383.2	**371.1**	375.4	0.29	<0.001***	
FDis		381.4	382.2	**378.2**	383.1	0.25	<0.001***	

Note

For each combination of predictor and response variable, we compared a simple linear model with models including UGS type and its interaction with the predictor, a quadratic term for the predictor, and both the interaction and the quadratic term. We defined as the best model the one with the smallest AICc (bold). *R* ^2^ and *p*‐values are only indicated for the best model.

* *p*<0.05 (0.01<*p*<0.05)

** *p*<0.01 (0.01<*p*<0.01)

*** *p*<0.01

## Supporting information

Appendix S1

Appendix S2

Appendix S3

## Data Availability

All data on total brood cells, parasitism level, and failure for each nest evaluated in the final analyses are included in the Appendices S1‐S3 accompanying this manuscript.
